# Depression and Anxiety Symptoms Among Combustible Cigarette Smokers, Heated Tobacco Users, and Non-Smokers: A Cross-Sectional Study in Lithuania

**DOI:** 10.3390/medicina62091618

**Published:** 2026-08-22

**Authors:** Liudas Vincentas Sinkevicius, Sandra Sakalauskaite, Karolis Rakauskas, Margus Lõokene, Rasa Pilkauskaite Valickiene, Danielius Serapinas

**Affiliations:** 1 Institute of Psychology, Mykolas Romeris University, 08303 Vilnius, Lithuaniad_serapinas@mruni.eu (D.S.); 2 Department of Health Psychology, Lithuanian University of Health Sciences, 50161 Kaunas, Lithuania; 3 Laboratory of Immunology, Department of Immunology and Allergology, Lithuanian University of Health Sciences, 50161 Kaunas, Lithuania; 4 Toompargi Vaimse Tervise Keskus, 10149 Tallinn, Estonia; 5 Department of Family Medicine, Medical Academy, Lithuanian University of Health Sciences, 50161 Kaunas, Lithuania

**Keywords:** addiction, anxiety, depression, GAD-7, HTPs, inflammation, nicotine, smoking, mental health, PHQ-9, public mental health

## Abstract

*Background and Objectives*: Tobacco smoking is associated with an increased risk of depression and anxiety symptoms, but it remains unclear whether this relationship differs according to the type of nicotine product used. The increasing popularity of heated tobacco products has created a need to better understand their association with mental health, particularly in comparison with combustible cigarettes. Therefore, this study aimed to compare the prevalence of depression and anxiety symptoms among combustible cigarette (CC) users, heated tobacco product (HTP) users, and non-smokers. *Materials and Methods*: A cross-sectional study was conducted among 497 adults. Participants were classified into three groups according to their tobacco-use status: combustible cigarette smokers, heated tobacco users, and non-smokers. Depressive symptoms were assessed using the Patient Health Questionnaire-9 (PHQ-9), and anxiety symptoms were assessed using the Generalized Anxiety Disorder-7 (GAD-7). Group differences were evaluated using hierarchical linear regression analyses adjusted for sociodemographic, health, and lifestyle factors. *Results*: Tobacco-use group significantly improved the model fit for PHQ-9 (ΔR^2^ = 0.0575, *p* < 0.001) and GAD-7 (ΔR^2^ = 0.0764, *p* < 0.001). In non-smokers, scores of depression were 3.48 lower than in CC users and 1.42 lower than in the HTP users’ group. In HTP users, these scores were 2.06 lower than in the CC users’ group. Anxiety scores were 3.71 lower in non-smokers than in CC users, and 1.29 lower than in the HTP users’ group. In the HTP users’ group, this score was 2.42 lower than in the CC users’ group. HC3 robust standard errors with 5000-sample BCa bootstrap confidence intervals and reduced-adjustment sensitivity analyses confirmed the primary pattern. *Conclusions*: Our study results showed that combustible cigarette users had the highest adjusted depression and anxiety scores, HTP users had intermediate scores, and non-smokers had the lowest. In both tobacco-using groups, greater product-specific nicotine dependence was associated with higher PHQ-9 and GAD-7 scores. Further interdisciplinary studies incorporating biomarkers of nicotine exposure, HPA axis function, inflammation, and oxidative stress are needed to clarify whether the observed differences reflect the characteristics of individuals who choose specific tobacco products or are partly attributable to product-specific effects.

## 1. Introduction

Tobacco use is one of the leading preventable causes of death and morbidity [1,2]. The relationship between cigarette smoking, nicotine dependence, and mental health has been extensively investigated, with numerous studies demonstrating associations with depression, anxiety, and other psychiatric outcomes [3,4]. Nicotine has a high potential for addiction: it acts on nicotinic acetylcholine receptors; activates the mesolimbic reward system; stimulates dopamine release; and repeated use leads to neuroadaptive changes, tolerance, craving, and withdrawal symptoms [5]. One possible mechanism linking nicotine dependence and mental health is dysregulation of the hypothalamic–pituitary–adrenal (HPA) axis. The HPA axis is the body’s main neuroendocrine system for responding to stress. During stress, corticotropin-releasing hormone, released by the hypothalamus, stimulates the secretion of adrenocorticotropic hormone by the pituitary gland, which in turn activates the release of cortisol from the adrenal cortex [6]. Nicotine can also activate this system: its action on nicotinic acetylcholine receptors stimulates the sympathetic nervous system and increases the concentrations of catecholamines and cortisol [7,8,9].

While acute nicotine exposure activates the HPA axis, chronic nicotine use has been associated with alterations in HPA-axis regulation and stress response. Long-term nicotine use can lead to neuroadaptive changes in the regulation and stress sensitivity of the HPA axis. Studies have indicated that nicotine use is associated not only with altered baseline cortisol levels but also with an altered response to psychological stressors. Evidence suggests that cortisol responses to psychological stress may be blunted in chronic smokers, and attenuated HPA-axis responses during early abstinence have been associated with withdrawal-related disturbances and an increased risk of smoking relapse [10,11]. There is also evidence linking HPA axis alterations to symptoms of depression and anxiety [12,13]. Chronic or repeated activation of stress response systems may be associated with impaired glucocorticoid receptor signaling, autonomic nervous system imbalance, sleep disturbances, and altered emotional regulation [13]. These processes may interact with inflammatory mechanisms. Impaired glucocorticoid receptor signaling may contribute to glucocorticoid resistance and insufficient regulation of inflammatory responses, thereby promoting chronic inflammation. Increased levels of pro-inflammatory cytokines may in turn affect the central nervous system, neurotransmitter metabolism, neuroplasticity, and brain areas involved in reward, threat assessment, and emotion regulation [14,15,16]. However, the relationship between nicotine use, the HPA axis, and mental health is not a single-component issue but rather a complex, multifactorial, and bidirectional mechanism; therefore, alterations in the HPA axis should be considered as one potential biological pathway rather than an established causal mechanism.

Nowadays, in addition to conventional combustible cigarettes, novel nicotine delivery products, such as heated tobacco products (HTPs), electronic cigarettes, and nicotine pouches, have become increasingly available. When assessing the effects of different nicotine products on the human body, it is important to distinguish the role of nicotine itself from the effects caused by substances produced during the combustion or heating of tobacco. Combustible cigarettes (CCs) produce large amounts of oxidative stress and inflammatory substances, such as carbon monoxide, tars, polycyclic aromatic hydrocarbons, particulate matter, aldehydes, and other compounds [17,18,19]. According to studies, the amounts of these substances in HTP aerosols are significantly lower due to the absence of a combustion process [20,21]. However, these still include substances such as glycerol and propylene glycol. The amount of nicotine in heated tobacco aerosol is approximately 70–80% of that found in cigarette smoke [22]. However, the actual exposure of users depends on: the product used, the depth of inhalation, the number of inhalations, and the frequency of use.

The comparison of exclusive combustible cigarette users with exclusive HTP users may provide descriptive evidence about whether mental health symptom patterns differ across tobacco products with different emission profiles. Naturally, a cross-sectional comparison cannot isolate the effects of nicotine from those of toxicants associated with tobacco combustion nor can it directly identify underlying biological mechanisms, as biomarkers of oxidative stress, inflammation, HPA-axis function, or toxicant exposure were not assessed. Nevertheless, differences in toxicant exposure between combustible cigarettes and HTPs provide a biological rationale for examining whether users of these products also differ in depressive and anxiety symptoms. If differences are observed, exposure to combustion-related toxicants and the potential effects on oxidative stress and inflammatory processes may be one of several possible explanations; however, this hypothesis cannot be tested directly with the present data.

Previous studies have shown that combustible cigarette use is associated with depressive and anxiety symptoms in a variety of populations. Current smokers show a 30–174% increased risk of depression, which was associated with a dose–response relationship compared with never smokers [4,23,24,25,26,27]. Former smokers also show an increased risk of depression [4], although to a lesser extent than current smokers, and smoking cessation has been associated with improvements in mental health [23,28,29,30]. However, data on HTPs are scarce, and the studies themselves are methodologically limited. Only a small number of studies have examined associations between HTP use and depressive or anxiety symptoms, while the methodological differences in study populations, definitions of product use, and mental health assessments limit direct comparisons across studies [31,32,33]. To our knowledge, evidence directly comparing depressive and anxiety symptoms among clearly defined exclusive HTP users, exclusive combustible cigarette smokers, and non-smokers remains limited. Therefore, this study aimed to compare the severity of depressive and anxiety symptoms among combustible cigarette smokers, HTP users, and non-smokers.

## 2. Materials and Methods

### 2.1. Participants and Procedure

A convenience sample was used to collect data for this study. During the study, an anonymous questionnaire survey was conducted in May–July 2026. An electronic questionnaire, compiled using the Google Forms internet platform, was distributed on social networks and in social network groups, such as Facebook and Instagram, through the researchers’ personal accounts. Prospective study participants were asked to complete the questionnaire and share it with friends. The questionnaire was distributed using the e-mail databases of Lithuania’s largest companies, including governmental institutions. Also, while conducting private training sessions in various organizations, the authors of the work asked separate groups, such as those who smoke or use HTPs, as well as those who do not use any nicotine products, to fill out a questionnaire. Only individuals aged 18 or older could participate in the study, but most were around 37 years old. The survey was expected to take up to 10 min to complete. The study participants were informed about the study and its purpose, confidentiality was ensured, and instructions for completing the survey and contact information were provided. In one of the first survey questions, respondents indicated which nicotine product they used (traditional cigarettes only, electronic cigarettes only, heated tobacco products only, or more than one nicotine product), and non-smokers were included as a control group. After selecting an answer, the survey automatically directed respondents to the part of the questionnaire adapted to them. For those who chose e-cigarettes or more than one nicotine product, the survey was ended; this ensured that each participant completed only the questions related to their chosen nicotine product, and that we collected only pure users of combustible cigarettes and heated tobacco. No formal a priori power calculation was performed; recruitment used a pragmatic target of approximately 150 participants per study group during the predefined data-collection period. A sensitivity calculation for the complete-case primary analysis (N = 473), with α = 0.05, 80% power, a two-parameter group block, and 10 total predictor parameters, indicated a minimum detectable incremental effect of f^2^ = 0.0205 (approximately 0.021). A total of 497 participants completed the online questionnaire, and this sample was split into three groups: 154 combustible cigarette users, 155 heated tobacco users, and a control group of 188 participants who reported not using any tobacco products (Table 1). Because the survey was anonymous, participants could not be contacted individually on the basis of their depression or anxiety responses; however, the final survey page provided contact information for free psychological-support helplines.

#### Demographic Variables

The participants were asked a wide range of demographic questions: gender, age, education, employment, income, frequency of alcohol consumption, use of psychoactive substances, sleep quality, and physical activity. These demographic factors were collected to be used as covariates. Participants in the tobacco-user groups also reported age at nicotine-use initiation; HTP users reported duration of HTP use. Nicotine dependence was assessed with product-specific instruments: the FTND among combustible cigarette users and the HeaTPAQ among HTP users. Because the two instruments differ in item contents and score metrics and are not calibrated to a common scale, their raw total scores were not used for direct between-product comparisons of dependence severity. The decision to use different nicotine-consumption instruments is based on the view that nicotine-containing products differ in their physiological effects, including the rate and mechanisms of nicotine absorption and consumption behavior [34,35]. These questionnaires were used for scientific research purposes only to assess expressions of general nicotine consumption, and not to determine physical dependence.

### 2.2. Questionnaires Used for Study

#### 2.2.1. The Fagerström Test for Nicotine Dependence Questionnaire

The Fagerström Test for Nicotine Dependence (FTND) was used for traditional cigarette users [34]. The Fagerström Nicotine Dependence Test (FTND) is a well-known and widely used instrument that helps to assess the strength of traditional cigarette-consumption behavior. This questionnaire is freely available electronically in Lithuanian; therefore, separate permission and translation for the use of this instrument for scientific purposes were not required. The FTND questionnaire was modified by Heatherton et al., which was based on the questionnaire previously developed by Fagerström (1978)—the Fagerström Tolerance Questionnaire (FTQ) [34]. The FTND scale consists of 6 ordinal questions (e.g., “When did you smoke your first cigarette after waking up in the morning?”), the answers to which were coded from 0 to 3, and a higher total score of the questionnaire indicates a higher manifestation of nicotine-use behavior. For this study, the overall internal consistency of the scale was calculated as Cronbach’s alpha = 0.77. This indicates that the internal consistency of the questionnaire in this study is satisfactory. In the literature, Cronbach’s alpha for the FTND ranges from 0.61 to 0.74 [34]. The authors of the FTND questionnaire and foreign studies support the validity of this instrument [34,36,37].

#### 2.2.2. The Heated Tobacco Product Addiction Questionnaire

The Heated Tobacco Product Addiction Questionnaire (HeaTPAQ) was developed by Feten Fekih-Romdhane and co-authors to assess specific aspects of addiction to heated tobacco products [38]. The Heated Tobacco Product Addiction Questionnaire (HeatPAQ) was used to assess heated tobacco product use, attitudes, and subjective evaluations. This instrument was designed to assess the frequency of use, signs of addiction, and perceived harm and satisfaction associated with heated tobacco products. The questionnaire consists of several questions covering consumption behavior and attitudes (e.g., “How often do you use heated tobacco products?” and “Do you think heated tobacco products are less harmful than regular cigarettes?”). Responses are assessed on a Likert-type scale, with higher total scores indicating more intensive use or more favorable attitudes towards heated tobacco. The questionnaire consists of 13 statements. Items were rated from 1 to 5 on a five-point Likert-type scale and summed, yielding a possible total score of 13 to 65; higher scores indicate a greater HTP dependence. No validated clinical cut-off categories were applied, and the score was analyzed as a continuous variable. Psychometric analyses showed the unifactorial structure and excellent internal consistency (Cronbach’s α = 0.90). Previous studies demonstrated sufficient internal consistency of this instrument, allowing it to be used in the study of tobacco-use-related behavior. Although the questionnaire is freely available, the authors were contacted by e-mail and consent to use it in the study was obtained. A double-blind translation into the Lithuanian language was also performed.

#### 2.2.3. The Generalized Anxiety Disorder-7 Scale

The Generalized Anxiety Disorder-7 (GAD-7) scale was used to assess the anxiety severity of the study participants. The GAD-7 scale was developed by Spitzer et al. (2006) and consists of 7 questions (e.g., “Excessive worry about various things”) [39]. The subjects were assessed using a Likert scale from 0 to 3, taking into account the frequency of anxiety over the past two weeks (0 = “not at all worried”, 1 = “a few days”, 2 = “more than half of all days”, and 3 = “almost every day”). The questionnaire was used for general self-assessment by respondents and not for establishing a clinical diagnosis. A higher total score on the questionnaire indicates a higher anxiety severity. The overall internal consistency of the scale was calculated as Cronbach’s alpha = 0.90. Similar results are reported in the literature (Cronbach’s alpha = 0.92) [39]. This questionnaire is freely available in electronic form in Lithuanian; therefore, no separate permission or translation was required to use this instrument for scientific purposes.

#### 2.2.4. The Patient Health Questionnaire-9

The Patient Health Questionnaire-9 (PHQ-9) was used to assess the severity of depressive symptoms among the study participants. This instrument is freely available and has been translated into Lithuanian, allowing its use for scientific purposes without additional permission. The PHQ-9 was developed by Kroenke et al. (2001) and consists of 9 items reflecting the diagnostic criteria for major depressive disorder (e.g., “Little interest or pleasure in doing things”) [40]. Participants rate how often they experienced each symptom over the past two weeks using a Likert scale from 0 to 3 (0 = “not at all”, 1 = “several days”, 2 = “more than half the days”, and 3 = “nearly every day”). The questionnaire was used as a self-report measure of depressive symptom severity and not for establishing a clinical diagnosis. A higher total score indicates greater depression severity. In the present study, the internal consistency of the scale was high (Cronbach’s alpha = 0.86). Previous studies have demonstrated excellent reliability of the depression, with Cronbach’s alpha typically around 0.86–0.89 [40].

### 2.3. Analysis Strategy

All statistical analyses were performed using jamovi 2.6.44 (the jamovi project, 2024), downloaded from [41]. Descriptive statistics were calculated for the full sample, and Spearman’s correlations were used to describe bivariate associations among study variables. Hierarchical linear regression analysis was used to assess the differences in depression (PHQ-9) and anxiety (GAD-7) scores between different groups of tobacco users and non-smokers. Sociodemographic and lifestyle covariates were entered in the first step of each regression model. In the second step, the tobacco-use group variable was added as a dummy variable, with cigarette users as the reference group. This approach allowed us to assess whether the tobacco-use group explained additional variance in depression and anxiety scores after controlling for the covariates. Changes in explained variance between the models were evaluated using ΔR^2^ and the corresponding F-change test. Given the nature of the variables analyzed, it was not expected that their distributions would fully comply with the assumption of normality. However, regression analysis was chosen because it is quite robust to violations of the normality assumption. Because income data were missing for 24 participants, the primary adjusted models were estimated using complete cases (N = 473). Product-specific dependence was examined in secondary within-group hierarchical models. Among combustible cigarette users, the FTND score was added to the same covariate block; among HTP users, the HeaTPAQ score was added to the same covariate block. These analyses used N = 146 and N = 151 because of missing income data. FTND and HeaTPAQ were not entered simultaneously in the three-group model, as each measure was structurally inapplicable outside its corresponding product group and the two scales are not calibrated to a common metric. Multicollinearity of predictors was assessed in all regression models using variance inflation factors (VIFs), condition indices, and variance-decomposition proportions. No significant multicollinearity was found in any of the models, as the variance inflation factor values were less than 4 (VIF < 4). The Q–Q plots of the standardized residuals were visually assessed. They showed some deviations from the normal distribution, especially in the extreme parts of the distributions. Therefore, sensitivity analyses were additionally performed using HC3 heteroskedasticity-consistent robust standard errors and bias-corrected and accelerated (BCa) 95% bootstrap confidence intervals based on 5000 bootstrap samples. PHQ-9 and GAD-7 scores were retained on their original validated scales and were not transformed. To assess possible over-adjustment, PHQ-9 and GAD-7 analyses were repeated without sleep quality and physical activity. A sensitivity analysis was also performed for the complete-case sample (N = 473). At α = 0.05 and 80% power, the minimum detectable incremental effect for the tobacco-use group was approximately f^2^ = 0.021.

## 3. Results

### 3.1. Participant Characteristics and Model Variables

#### Descriptive Statistics of the Study Sample

Descriptive statistics for the study variables are presented in Table 2. The study included 497 individuals who were divided into three groups based on the type of tobacco product they used: (i) combustible cigarette (CC) users; (ii) heated tobacco product (HTP) users; and (iii) non-smokers. A total of 263 participants were female (52.9%), and 234 were male (47.1%). The gender distribution was balanced across the three study groups: women accounted for 52.6% of CC users, 50.3% of HTP users, and 55.3% of non-smokers. The mean age ranged from 35.5 years among CC users, 36.1 years among HTP users, and 39.3 years among non-smokers, indicating that the study sample was predominantly working-age adults. Most participants were employed full-time, and the sample predominantly comprised working-age adults. These characteristics describe the convenience sample reached by the recruitment strategy and should not be interpreted as evidence of population representativeness. The distribution of education between groups was also broadly similar: most participants reported secondary, vocational, college, or university education.

Income data were available for 473 participants and showed some differences between groups: CC users reported slightly higher mean income category scores than HTP users or non-smokers. Differences were also observed on several lifestyle variables. CC users reported the highest mean frequency of alcohol consumption, HTP users reported intermediate values, and non-smokers reported the lowest values. A similar gradual pattern was observed for sleep quality: CC users reported the lowest mean score, HTP users reported intermediate values, and non-smokers reported the highest. Physical activity scores were relatively similar across the three groups, although the mean score for HTP users was slightly lower than for CC users and non-smokers. Despite the aforementioned group differences, these three groups were sufficiently similar in their baseline sociodemographic characteristics to allow for further group analyses. At the same time, the observed differences in age, income, alcohol consumption frequency, sleep quality, and physical activity warranted their inclusion as covariates in the hierarchical regression models. It should also be noted that the income analyses were based on a slightly smaller sample due to missing responses. In contrast, the remaining descriptive analyses were based on the full sample.

### 3.2. Hierarchical Regression Analysis

#### 3.2.1. Depression Scores

Hierarchical linear regression analysis showed that the first model, which included sociodemographic and lifestyle covariates, was statistically significant, F(8, 464) = 29.3, *p* < 0.001, and explained 33.5% of the variance in depression scores (R^2^ = 0.335; adjusted R^2^ = 0.324) (see Table 3). When the tobacco-use group variable was additionally included in the second model, the proportion of explained variance increased to 39.3% (R^2^ = 0.393; adjusted R^2^ = 0.380), and the overall model remained statistically significant, F(10, 462) = 29.9, *p* < 0.001. The inclusion of tobacco-use group significantly improved the model, ΔR^2^ = 0.0575, ΔF(2, 462) = 21.9, *p* < 0.001.

In the final model, depression scores were statistically significantly associated with sex (B = −2.2599, β = −0.2295, *p* < 0.001), employment (B = 0.4846, β = 0.1071, *p* = 0.008), sleep quality (B = −1.4994, β = −0.3113, *p* < 0.001), and physical activity (B = −0.5293, β = −0.1147, *p* = 0.003). Age, education, income, and alcohol frequency were not statistically significant predictors of depression scores (all *p* > 0.05), although alcohol frequency approached statistical significance (B = 0.3387, β = 0.0759, *p* = 0.054). After controlling for the other variables in the model, adjusted PHQ-9 scores were highest among CC users. HTP users scored on average 2.06 points lower than CC users (B = −2.0644, β = −0.4190, *p* < 0.001), whereas non-users scored 3.48 points lower than CC users (B = −3.4811, β = −0.7066, *p* < 0.001) and 1.42 points lower than HTP users (B = −1.4167, β = −0.2876, *p* = 0.002).

#### 3.2.2. Anxiety Scores

In Table 4, the first hierarchical regression model for anxiety was statistically significant, F(8, 464) = 25.1, *p* < 0.001, and explained 30.2% of the variance in anxiety scores (R^2^ = 0.302; adjusted R^2^ = 0.290). When the group variable was included, the final model explained 37.8% of the variance in GAD-7 scores (R^2^ = 0.378; adjusted R^2^ = 0.365) and was statistically significant, F(10, 462) = 28.1, *p* < 0.001. The inclusion of group significantly increased the explanatory power of the model, ΔR^2^ = 0.0764, ΔF(2, 462) = 28.4, *p* < 0.001.

In the final model, lower anxiety scores were statistically significantly associated with higher age (B = −0.0508, β = −0.1133, *p* = 0.005), gender (B = −2.0226, β = −0.2212, *p* < 0.001), sleep quality (B = −1.1836, β = −0.2647, *p* < 0.001), and physical activity (B = −0.4483, β = −0.1046, *p* = 0.007). Employment was positively associated with anxiety scores (B = 0.3591, β = 0.0854, *p* = 0.036). Education, income, and frequency of alcohol consumption were not statistically significant predictors (*p* > 0.05). After controlling other variables in the model, anxiety scores were highest in the CC users’ group. Anxiety scores for non-smokers were 3.71 points lower than those in CC users (B = −3.7135, β = −0.8118, *p* < 0.001) and 1.29 points lower than those in HTP users (B = −1.2896, β = −0.2819, *p* = 0.002). In the group of HTP users, anxiety scores were on average 2.42 points lower than those in the CC users’ group (B = −2.4239, β = −0.5299, *p* < 0.001).

#### 3.2.3. Nicotine Dependence Analyses

Product-specific hierarchical analyses were conducted within the two tobacco-user groups (Table 5). Among combustible cigarette users, adding the FTND score significantly improved the PHQ-9 model (ΔR^2^ = 0.0917, ΔF(1, 136) = 21.7, *p* < 0.001) and the GAD-7 model (ΔR^2^ = 0.0547, ΔF(1, 136) = 12.1, *p* < 0.001). Higher FTND scores were associated with higher PHQ-9 scores (B = 0.930, SE = 0.200, β = 0.395, *p* < 0.001) and higher GAD-7 scores (B = 0.679, SE = 0.196, β = 0.305, *p* < 0.001). Among HTP users, adding the HeaTPAQ score significantly improved both the PHQ-9 model (ΔR^2^ = 0.0429, ΔF(1, 141) = 8.53, *p* = 0.004) and the GAD-7 model (ΔR^2^ = 0.0470, ΔF(1, 141) = 8.38, *p* = 0.004). Higher HeaTPAQ scores were associated with higher PHQ-9 scores (B = 0.107, SE = 0.037, β = 0.271, *p* = 0.004) and higher GAD-7 scores (B = 0.100, SE = 0.035, β = 0.284, *p* = 0.004). These within-product analyses assess graded associations with dependence severity but do not provide a direct cross-product comparison of dependence because FTND and HeaTPAQ scores are not on a common metric.

#### 3.2.4. Model-Diagnostic and Robustness Sensitivity Analyses

Additional diagnostic and sensitivity analyses were performed to assess the robustness of the primary findings. Multicollinearity diagnostics did not indicate a problematic predictor structure, as all VIF values were below four, and the maximum condition index in the complete-case predictor matrix (N = 473) was 2.64, with no substantial variance-decomposition proportions shared by multiple predictors.

Because some deviations were observed in the residual distribution, particularly in the tails, the primary group effects were re-estimated using HC3 robust standard errors and 5000-sample BCa bootstrap confidence intervals. The tobacco-use group remained significantly associated with both PHQ-9 (robust F(2, 462) = 16.37, *p* < 0.001) and GAD-7 scores (robust F(2, 462) = 19.33, *p* < 0.001). For PHQ-9, HTP users scored 2.06 points lower than CC smokers (B = −2.064, 95% BCa CI [−3.204, −0.947], *p* < 0.001), while the difference between non-users and CC smokers was 3.48 points (B = −3.481, 95% BCa CI [−4.658, −2.337], *p* < 0.001). Non-users also had lower PHQ-9 scores than HTP users (B = −1.417, 95% BCa CI [−2.256, −0.626], *p* < 0.001). The same ordering was observed for GAD-7: compared with CC smokers, scores were lower among HTP users (B = −2.424, 95% BCa CI [−3.517, −1.346], *p* < 0.001) and non-users (B = −3.713, 95% BCa CI [−4.899, −2.587], *p* < 0.001). Non-users also scored lower than HTP users (B = −1.290, 95% BCa CI [−2.052, −0.553], *p* = 0.001).

Dependence severity was positively associated with depressive and anxiety symptoms within both tobacco-use groups. Among CC smokers, higher FTND scores were associated with higher PHQ-9 (B = 0.930, β = 0.395, 95% BCa CI [0.500, 1.393], *p* < 0.001) and GAD-7 scores (B = 0.679, β = 0.305, 95% BCa CI [0.303, 1.138], *p* = 0.003). A similar pattern was found among HTP users, with higher HeaTPAQ scores associated with PHQ-9 (B = 0.107, β = 0.271, 95% BCa CI [0.040, 0.168], *p* = 0.002) and GAD-7 scores (B = 0.100, β = 0.284, 95% BCa CI [0.036, 0.160], *p* = 0.002).

A further sensitivity analysis examined whether adjustment for sleep quality and physical activity influenced the between-group findings. When these variables were excluded, adding the tobacco-use group increased the explained variance in PHQ-9 scores from 16.0% to 28.9% (ΔR^2^ = 0.129, ΔF(2, 464) = 42.1, *p* < 0.001). Compared with CC smokers, PHQ-9 scores were 3.19 points lower among HTP users (B = −3.187, *p* < 0.001) and 4.85 points lower among non-users (B = −4.850, *p* < 0.001). For GAD-7, the explained variance increased from 15.2% to 30.1% after the tobacco-use group was added (ΔR^2^ = 0.148, ΔF(2, 464) = 49.2, *p* < 0.001). Again, CC smokers had the highest scores, with HTP users scoring 3.30 points lower (B = −3.305, *p* < 0.001) and non-users 4.80 points lower (B = −4.796, *p* < 0.001). The group differences therefore remained significant when sleep quality and physical activity were excluded from the models.

## 4. Discussion

Our study is one of the few studies that examines and compares the distinct tobacco products associations with mental health. We found that individuals who used combustible cigarettes had the highest estimates of both psychological indicators. After adjustment, HTP users scored 2.06 points lower than CC users on the PHQ-9 and 2.42 points lower on the GAD-7, whereas non-users scored 3.48 and 3.71 points lower than CC users, respectively. Non-users also had lower scores than HTP users on both outcomes. The use of heated tobacco products as the comparison group represents one of the strengths of the present study, as it allowed an indirect evaluation of the hypothesis that combustion-related toxicants rather than nicotine alone may contribute to depressive and anxiety symptoms. Although the present study did not assess biological markers, the observed gradient between CC smokers and HTP users are compatible with the hypothesis that reduced exposure to combustion-related toxicants may result in lower oxidative stress and inflammatory activity, potentially contributing to lower levels of depressive and anxiety symptoms.

Our findings are in agreement with previous longitudinal evidence demonstrating an association between cigarette smoking and depressive and anxiety symptoms and previous cross-sectional studies showing poorer mental health among smokers than among non-smokers [42,43]. Evidence regarding HTP use, however, remains limited and heterogeneous. Unlike most previous studies, which evaluated smokers as a single group or focused primarily on combustible cigarettes, our study directly compared CC smokers with HTP users, with non-smokers included as a reference group, and observed differences in depressive and anxiety symptom severity between the groups. Similar, to Lee et al., we reported that users of nicotine products, including HTPs, had a higher prevalence of depressive symptoms than non-users [33]. However, Lee et al. found that HTP-only users had a higher prevalence of moderate-to-severe depressive symptoms than CC-only users and, after adjustment, showed a stronger association with moderate-to-severe depressive symptoms relative to non-users. This differs from the pattern observed in our study, in which depressive symptom scores were higher among CC smokers than among HTP users. Differences in participant characteristics, outcome definitions, adjustment strategies, and tobacco-use patterns may explain these discrepant findings. Cho et al. [44] reported that both combustible cigarette smokers and users of non-combustible nicotine or tobacco products had higher odds of generalized anxiety disorders than never-users, with numerically higher odds among combustible cigarette smokers. However, their non-combustible group was broader than HTP use alone, and the study focused specifically on generalized anxiety disorder rather than depressive and anxiety symptom severity. In contrast, our study directly compared exclusive CC and HTP users using continuous PHQ-9 and GAD-7 scores. Overall, differences in study populations, definitions of tobacco product use, mental health measures, and covariate adjustment may contribute to the heterogeneous findings across studies.

The characteristics of the subjects in our study provide context for the interpretation of the results. All three groups had similar numbers of participants, and the distribution of women and men was relatively balanced. The mean age across the groups ranged from approximately 35.5 to 39.3 years, reflecting that most subjects were middle-aged adults. The majority of participants in all groups were employed full-time. This suggests that the results are not based exclusively on data from the unemployed, students, retirees, or clinical populations, but are largely representative of socially and economically active adults engaged in daily working lives. The groups were also relatively similar in terms of educational attainment, and general employment profile. This similarity reduced the likelihood that the differences in depression and anxiety were explained solely by the strong sociodemographic distinctiveness of one group, like in Seo et al. (2024)’s research [45]. In addition, the regression models statistically controlled for age, sex, education, employment, and income. However, the groups were not completely identical. The non-user group was slightly older, the combustible cigarette user group had a higher mean frequency of alcohol consumption, and sleep quality scores also differed between the groups. For this reason, adjusted regression models are more informative than a simple comparison of unadjusted means. Even after controlling sociodemographic, lifestyle, and health factors, the inclusion of the group variable statistically significantly improved the explanatory power of both regression models. In the depression model, the group variable explained an additional 5.75% of the variance in depression, and 7.64% of the variance in anxiety model. These group associations remained statistically significant when inference was based on HC3 robust standard errors and BCa bootstrap confidence intervals. Thus, tobacco-use group remained associated with symptom severity after statistical adjustment. This indicates that the tobacco-use group was associated with depressive and anxiety symptomatology after controlling for age, gender, education, employment, income, quality of life, physical activity, and other confounders included in the models, but ΔR^2^ should be interpreted as an incremental association rather than a causal contribution.

We found that depression and anxiety scores were associated with gender, employment, sleep quality, and physical activity, but were not related to education or income. In addition, anxiety scores were significantly associated with age. Similar trends were also shown in a recent national sample study conducted in Korea, in which both exclusive users of heated tobacco and users of both combustible cigarettes and heated tobacco were more likely to have depressive symptoms than non-smokers [33].

According to our study, CC users had the highest scores on both psychological indicators. Compared to this group, HTP users had an average of 2.06 points lower depression scores and 2.42 points lower anxiety scores. Even greater differences were found when comparing CC users with non-smokers: non-smokers had a depression score of 3.48 points lower and an anxiety score of 3.71 point lower. Thus, in the non-smokers group, both scales’ scores were significantly lower than those of combustible tobacco users. This structure of the results is consistent with the study hypothesis that the combustion process and the associated higher exposure to toxic compounds may be one of the factors linking cigarette use to poorer psychological well-being. Tobacco combustion produces many reactive compounds that can promote oxidative stress and inflammatory processes [46,47]. These biological processes could theoretically affect neurotransmitter metabolism, neuroendocrine regulation, mitochondrial function, and other mechanisms related to symptoms of depression and anxiety [48]. Since HTPs do not burn in the same way as combustible cigarettes [49], the lower depression and anxiety scores in this group could be related to lower exposure to toxic compounds and the potential oxidative stress that this may cause, although this hypothesis requires confirmation in longitudinal studies incorporating biomarkers of oxidative stress, inflammation, HPA-axis activity, and nicotine exposure.

Although the lowest scores for depression and anxiety symptoms were found in the group of non-users of tobacco products, a proportion of them also had symptoms of depression and anxiety. This result is not unexpected and is consistent with epidemiological studies [50] showing that symptoms of depression and anxiety are relatively common in the general population and reminds that mental health is determined by many interacting factors, and smoking is only one of the possible risk factors. Mental health is determined by the complex interaction of biological, psychological, and social factors, including genetic susceptibility, chronic stress, sleep disturbances, physical inactivity, social environment, and coexisting health conditions [51,52]. Our regression models showed this: poorer sleep quality and lower physical activity were associated with higher depression and anxiety scores.

The product-specific dependence analyses provide additional context for the observed differences between tobacco-use groups. Greater dependence severity was associated with higher depressive and anxiety symptom scores within both groups: higher FTND scores were associated with higher PHQ-9 and GAD-7 scores among CC smokers, while similar associations were observed for HeaTPAQ scores among HTP users. However, FTND and HeaTPAQ are product-specific instruments with different scoring systems and cannot be directly compared between groups. Therefore, although dependence severity was related to mental health symptoms within both groups, the present analyses cannot determine whether differences in dependence contribute to the observed differences in depressive and anxiety symptoms between CC and HTP users.

Sleep quality and physical activity also require careful consideration. These variables were included as potential lifestyle confounders, but they may themselves be related to nicotine use and to depressive and anxiety symptoms. Their inclusion in the primary models could therefore have attenuated the associations with tobacco-use group. However, sensitivity analyses excluding sleep quality and physical activity showed the same overall pattern, with the differences between tobacco-use groups becoming somewhat larger. This suggests that the observed group differences were not dependent on adjustment for these factors. Nevertheless, the cross-sectional design does not allow the direction of these associations to be established. Depressive and anxiety symptoms may themselves influence continued smoking, cessation difficulty, switching between products, or the choice of tobacco product. Reverse causation therefore remains a plausible alternative explanation for the observed associations.

Several limitations should be considered when interpreting these results. First, the cross-sectional design precludes the establishment of causal relationships, and reverse causality remains a possibility, as symptoms of depression or anxiety could influence smoking persistence, cessation, switching behavior, or product choice. Although several sociodemographic, health, and lifestyle factors were included in the analysis, residual confounding cannot be ruled out regarding factors such as mental health history, socioeconomic stressors, or motivations for product choice. Second, convenience-based online recruitment may have introduced selection and non-response biases, limiting the generalizability of the findings. Furthermore, including only single-product users in the study may not fully reflect real-world patterns, given the prevalence of dual users; however, the study’s objective necessitated this approach. Finally, the observed differences in depressive and anxiety symptoms cannot be attributed to oxidative stress or other proposed biological mechanisms, since biomarkers of oxidative stress, inflammation, HPA-axis function, or toxicant exposure were not assessed. Therefore, the biological mechanism remains theoretical and should be tested in future studies, including measures such as oxidative damage, the antioxidant system, and systemic inflammation.

Our study results highlight the need for a more interdisciplinary approach to understanding the links between different patterns of tobacco product use and mental health. Although research to date has mostly focused on individual biological or psychosocial aspects of this relationship, comprehensive studies that integrate knowledge from medicine, psychology, public health, and social sciences are still limited. Future research combining medical, psychological, public health, and social science approaches could simultaneously assess potential biological pathways, including HPA-axis regulation, inflammation, and oxidative stress, together with psychological, behavioral, and social factors related to tobacco use and mental health. Such an integrated approach may help clarify the complex and potentially bidirectional relationships observed in the present study and provide a stronger basis for future prevention and intervention strategies.

## 5. Conclusions

Our study results showed that combustible cigarette users had the highest adjusted depression and anxiety scores, HTP users had intermediate scores, and non-smokers had the lowest. In both tobacco-using groups, greater product-specific nicotine dependence was associated with higher PHQ-9 and GAD-7 scores. Further interdisciplinary studies incorporating biomarkers of nicotine exposure, HPA-axis function, inflammation, and oxidative stress are needed to clarify whether the observed differences reflect the characteristics of individuals who choose specific tobacco products or are partly attributable to product-specific effects.

## Figures and Tables

**Table 1:** Participant characteristics by tobacco-use group.

Characteristic	Total Sample (N = 497)	Cigarette Users (n = 154)	Heated Tobacco Users (n = 155)	Non-Smokers (n = 188)
Age, years, M (SD)	37.12 (10.44)	35.50 (10.20)	36.10 (9.26)	39.30 (11.20)
Sex, N (%)				
Women	263 (52.9)	81 (52.6)	78 (50.3)	104 (55.3)
Men	234 (47.1)	73 (47.4)	77 (49.7)	84 (44.7)
Education, N (%)				
Basic	4 (0.8)	2 (1.3)	0 (0.0)	2 (1.1)
Secondary	34 (6.8)	17 (11.0)	9 (5.8)	8 (4.3)
Vocational	113 (22.7)	39 (25.3)	33 (21.3)	41 (21.8)
College	146 (29.4)	47 (30.5)	51 (32.9)	48 (25.5)
Higher education	200 (40.2)	49 (31.8)	62 (40.0)	89 (47.3)
Monthly income, N (%)				
≤€800	38 (8.0)	9 (6.2)	13 (8.6)	16 (9.1)
€800–1200	139 (29.4)	27 (18.5)	43 (28.5)	69 (39.2)
€1201–1600	120 (25.4)	28 (19.2)	46 (30.5)	46 (26.1)
€1601–2200	111 (23.5)	54 (37.0)	31 (20.5)	26 (14.8)
≥€2201	65 (13.7)	28 (19.2)	18 (11.9)	19 (10.8)
Employment status, N (%)				
Full-time employment	353 (71.0)	108 (70.1)	111 (71.6)	134 (71.3)
Part-time employment	63 (12.7)	16 (10.4)	21 (13.5)	26 (13.8)
Student	45 (9.1)	17 (11.0)	14 (9.0)	14 (7.4)
Employed and studying	14 (2.8)	5 (3.2)	6 (3.9)	3 (1.6)
Unemployed	14 (2.8)	4 (2.6)	3 (1.9)	7 (3.7)
Retired	6 (1.2)	3 (1.9)	0 (0.0)	3 (1.6)
Maternity/parental leave	2 (0.4)	1 (0.6)	0 (0.0)	1 (0.5)
Age at nicotine-use initiation, years, M (SD)	17.00 (3.93)	16.70 (4.03)	17.30 (3.82)	—
Duration of heated tobacco use, years, M (SD)	2.79 (0.46)	—	2.79 (0.46)	—
Alcohol-use frequency, M (SD)	1.86 (1.11)	2.31 (1.16)	1.94 (1.09)	1.43 (0.91)
Sleep quality, M (SD)	3.13 (1.02)	2.51 (1.01)	3.32 (0.87)	3.49 (0.92)
Physical activity, M (SD)	1.42 (1.08)	1.45 (1.09)	1.28 (1.06)	1.50 (1.08)

Note. Values are M (SD) for scale scores and N (%) for categorical variables. Percentages are calculated within each tobacco-use group. Income data were available for 473 participants (cigarette users, N = 146; heated tobacco users, N = 151; non-smokers, N = 176). Age of nicotine-use initiation was available only for current tobacco users (N = 309). Duration of heated tobacco use was applicable only to heated tobacco users (n = 155). An em dash indicates that the variable was not applicable.

**Table 2:** Descriptive statistics and correlations among study variables.

Variable	Missing	M	SD	Skewness	Kurtosis	1	2	3	4	5	6	7	8	9	10	11
Combustible cigarettes	343	5.67	2.45	−0.73	−0.30	—										
Heated tobacco	342	39.94	9.93	−0.38	−0.50	—	—									
Anxiety	0	5.20	4.55	1.43	1.77	0.465 ***	0.432 ***	—								
Depression	0	5.72	4.93	1.32	1.50	0.512 ***	0.411 ***	0.818 ***	—							
Age	0	37.13	10.45	0.54	−0.33	−0.077	−0.010	−0.199 ***	−0.153 ***	—						
Education	0	4.01	0.99	−0.66	−0.46	−0.062	−0.053	−0.052	−0.041	0.296 ***	—					
Employment	0	1.59	1.14	2.25	4.98	−0.068	−0.209 **	0.084	0.114 *	−0.303 ***	−0.200 ***	—				
Income	24	3.05	1.18	0.10	−0.97	0.204 *	−0.119	0.060	0.133 **	0.250 ***	0.353 ***	−0.383 ***	—			
Alcohol frequency	0	1.86	1.11	0.38	−0.12	0.178 *	0.003	0.219 ***	0.213 ***	0.030	0.028	−0.092 *	0.179 ***	—		
Sleep quality	0	3.13	1.02	−0.10	−0.42	−0.574 ***	−0.482 ***	−0.462 ***	−0.470 ***	0.170 ***	0.223 ***	0.002	−0.035	−0.167 ***	—	
Physical activity	0	1.42	1.08	0.24	−0.56	−0.109	−0.435 ***	−0.143 ***	−0.171 ***	−0.023	0.056	0.043	0.077	−0.112 *	0.214 ***	—

Note. M = mean; SD = standard deviation. Correlations are Spearman’s rho. * *p* < 0.05, ** *p* < 0.01, *** *p* < 0.001. The correlation between combustible cigarette and heated tobacco scores was unavailable because there were no paired valid observations.

**Table 3:** Hierarchical regression analysis predicting depression total score.

Predictor	Estimate	SE	t	*p*	Stand. Estimate
Intercept	15.3843	1.3667	11.257	<0.001	
Age	−0.0265	0.0193	−1.373	0.171	−0.0548
Sex	−2.2599	0.3732	−6.055	<0.001	−0.2295
Education	−0.0535	0.2078	−0.257	0.797	−0.0106
Employment	0.4846	0.1816	2.669	0.008	0.1071
Income	0.3198	0.1843	1.735	0.083	0.0768
Alcohol frequency	0.3387	0.1755	1.930	0.054	0.0759
Sleep quality	−1.4994	0.2042	−7.343	<0.001	−0.3113
Physical activity	−0.5293	0.1769	−2.992	0.003	−0.1147
Group:					
2–1	−2.0644	0.5027	−4.107	<0.001	−0.4190
3–1	−3.4811	0.5262	−6.616	<0.001	−0.7066
3–2	−1.4167	0.4477	−3.164	0.002	−0.2876

Note. Final model: R^2^ = 0.393, adjusted R^2^ = 0.380, F(10, 462) = 29.9, *p* < 0.001, N = 473. When compared with Model 1: ΔR^2^ = 0.0575, ΔF(2, 462) = 21.9, *p* < 0.001. The intercept represents the reference level; pairwise group comparisons are presented. Group was treated as a categorical variable: Group 1 = CC users; Group 2 = HTP users; and Group 3 = non-users of tobacco products. Conventional OLS coefficients are shown; HC3/BCa sensitivity results are reported in Section 3.2.4.

**Table 4:** Hierarchical regression analysis predicting GAD-7 total score.

Predictor	Estimate	SE	t	*p*	Stand. Estimate
Intercept	15.5151	1.2842	12.081	<0.001	
Age	−0.0508	0.0181	−2.805	0.005	−0.1133
Sex	−2.0226	0.3507	−5.768	<0.001	−0.2212
Education	0.0867	0.1953	0.444	0.657	0.0185
Employment	0.3591	0.1707	2.104	0.036	0.0854
Income	−0.0495	0.1732	−0.286	0.775	−0.0128
Alcohol frequency	0.2214	0.1649	1.343	0.180	0.0534
Sleep quality	−1.1836	0.1919	−6.169	<0.001	−0.2647
Physical activity	−0.4483	0.1662	−2.697	0.007	−0.1046
Group:					
2–1	−2.4239	0.4723	−5.132	<0.001	−0.5299
3–1	−3.7135	0.4945	−7.510	<0.001	−0.8118
3–2	−1.2896	0.4207	−3.065	0.002	−0.2819

Note. Final model: R^2^ = 0.378, adjusted R^2^ = 0.365, F(10, 462) = 28.1, *p* < 0.001, N = 473. When compared with Model 1, ΔR^2^ = 0.0764, ΔF(2, 462) = 28.4, *p* < 0.001. The intercept represents the reference level; pairwise group comparisons are presented. Group was treated as a categorical variable: Group 1 = CC users; Group 2 = HTP users; and Group 3 = non-smokers. Conventional OLS coefficients are shown; HC3/BCa sensitivity results are reported in Section 3.2.4.

**Table 5:** Product-specific dependence sensitivity analyses for PHQ-9 and GAD-7 scores.

Group	Outcome	N	R^2^ Model 1 → Final	ΔR^2^	ΔF	B (SE)	β	*p*
CC users	PHQ-9	146	0.334 → 0.426	0.0917	21.7	0.930 (0.200)	0.395	<0.001
CC users	GAD-7	146	0.328 → 0.383	0.0547	12.1	0.679 (0.196)	0.305	<0.001
HTP users	PHQ-9	151	0.249 → 0.291	0.0429	8.53	0.107 (0.037)	0.271	0.004
HTP users	GAD-7	151	0.162 → 0.209	0.0470	8.38	0.100 (0.035)	0.284	0.004

Note. Model 1 included age, sex, education, employment, income, alcohol-use frequency, sleep quality, and physical activity. The final model added the product-specific dependence score. For CC models, ΔF df = (1, 136); for HTP models, ΔF df = (1, 141). CC = combustible cigarette; FTND = Fagerström Test for Nicotine Dependence; HeaTPAQ = Heated Tobacco Product Addiction Questionnaire; HTP = heated tobacco product. Conventional hierarchical OLS results are shown; HC3/BCa sensitivity results are reported in Section 3.2.4.

## Data Availability

The data supporting the findings of this study are available from the corresponding author upon reasonable request.

## Abbreviations

The following abbreviations are used in this manuscript:

GAD-7	Generalized Anxiety Disorder-7 scale
HPA	Hypothalamus–Pituitary–Adrenal axis
HTP	Heated Tobacco Product
PHQ-9	The Patient Health Questionnaire-9

## References

[1] Tobacco and Nicotine. https://www.who.int/news-room/fact-sheets/detail/tobacco.

[2] Goodchild M., Nargis N., Tursan d’Espaignet E. (2018). Global economic cost of smoking-attributable diseases. Tob. Control.

[3] Morissette S.B., Tull M.T., Gulliver S.B., Kamholz B.W., Zimering R.T. (2007). Anxiety, anxiety disorders, tobacco use, and nicotine: A critical review of interrelationships. Psychol. Bull..

[4] Hu Z., Cui E., Chen B., Zhang M. (2025). Association between cigarette smoking and the risk of major psychiatric disorders: A systematic review and meta-analysis in depression, schizophrenia, and bipolar disorder. Front. Med..

[5] Benowitz N.L. (2010). Nicotine addiction. N. Engl. J. Med..

[6] Herman J.P., McKlveen J.M., Ghosal S., Kopp B., Wulsin A., Makinson R., Scheimann J., Myers B. (2016). Regulation of the hypothalamic-pituitary-adrenocortical stress response. Compr. Physiol..

[7] Changeux J.P. (2010). Nicotine addiction and nicotinic receptors: Lessons from genetically modified mice. Nat. Rev. Neurosci..

[8] Sansone L., Milani F., Fabrizi R., Belli M., Cristina M., Zagà V., de Iure A., Cicconi L., Bonassi S., Russo P. (2023). Nicotine: From discovery to biological effects. Int. J. Mol. Sci..

[9] Sinkevicius L.V., Sakalauskaite S., Bukovskis M., Lõokene M., Valvere V., Gradauskiene B., Viigimaa M. (2025). Nicotine from a different angle: Biological effects from a psychoneuroimmunological perspective. Int. J. Mol. Sci..

[10] Richards J.M., Stipelman B.A., Bornovalova M.A., Daughters S.B., Sinha R., Lejuez C.W. (2011). Biological mechanisms underlying the relationship between stress and smoking: State of the science and directions for future work. Biol. Psychol..

[11] LaFond M., DeAngelis B., al’Absi M. (2024). Hypothalamic pituitary adrenal and autonomic nervous system biomarkers of stress and tobacco relapse: Review of the research. Biol. Psychol..

[12] Gold P.W. (2015). The organization of the stress system and its dysregulation in depressive illness. Mol. Psychiatry.

[13] Pariante C.M., Lightman S.L. (2008). The HPA axis in major depression: Classical theories and new developments. Trends Neurosci..

[14] Haroon E., Raison C.L., Miller A.H. (2012). Psychoneuroimmunology meets neuropsychopharmacology: Translational implications of the impact of inflammation on behavior. Neuropsychopharmacology.

[15] Dantzer R., O’Connor J.C., Freund G.G., Johnson R.W., Kelley K.W. (2008). From inflammation to sickness and depression: When the immune system subjugates the brain. Nat. Rev. Neurosci..

[16] Miller G.E., Cohen S., Ritchey A.K. (2002). Chronic psychological stress and the regulation of pro-inflammatory cytokines: A glucocorticoid-resistance model. Health Psychol..

[17] Haleem A.M., Amin S., Mahmood U.H. (2020). Heavy metal and polycyclic aromatic hydrocarbons in cigarettes: An analytical assessment. Popul. Med..

[18] Vu A.T., Taylor K.M., Holman M.R., Ding Y.S., Hearn B., Watson C.H. (2015). Polycyclic aromatic hydrocarbons in the mainstream smoke of popular U.S. cigarettes. Chem. Res. Toxicol..

[19] Adesina O.A., Olowolafe T.I., Igbafe A. (2022). Levels of polycyclic aromatic hydrocarbon from mainstream smoke of tobacco products and its risks assessment. J. Hazard. Mater. Adv..

[20] Kanobe M.N., Dull G.M., Darnell J., Jin T., Brown B., Coffield J., Keyser B.M., Fearon I.M., Makena P., Baxter S.A. (2024). Evaluation of environmental emissions from glo heated tobacco products and combustible cigarettes. Environ. Adv..

[21] Sakalauskaite S., Zdanavicius L., Šteinmiller J., Istomina N. (2025). Exposure to toxic compounds using alternative smoking products: Analysis of empirical data. Int. J. Environ. Res. Public Health.

[22] Jankowski M., Brożek G.M., Lawson J., Skoczyński S., Majek P., Zejda J.E. (2019). New ideas, old problems? Heated tobacco products—A systematic review. Int. J. Occup. Med. Environ. Health.

[23] Wu Z., Yue Q., Zhao Z., Wen J., Tang L., Zhong Z., Yang J., Yuan Y., Zhang X. (2023). A cross-sectional study of smoking and depression among US adults: NHANES (2005–2018). Front. Public Health.

[24] Bandiera F.C., Arguelles W., Gellman M., Castañeda S.F., Barnhart J., Gonzalez P., Navas-Nacher E.L., Salgado H., Talavera G.A., Schneiderman N. (2015). Cigarette smoking and depressive symptoms among Hispanic/Latino adults: Results from the Hispanic Community Health Study/Study of Latinos (HCHS/SOL). Nicotine Tob. Res..

[25] Lu Y., Chen H., Gan J., Cai J., Huang C., Chen Q. (2025). Depression, smoking, and lung cancer vulnerability: Bridging mental-physical comorbidity through population-based evidence. Tob. Induc. Dis..

[26] Tomioka K., Shima M., Saeki K. (2021). Association between heaviness of cigarette smoking and serious psychological distress is stronger in women than in men: A nationally representative cross-sectional survey in Japan. Harm Reduct. J..

[27] Huang J., Shi P., Zhao Y., Zhang H., Gao T., Wang X. (2024). Associations between smoking, sex steroid hormones, trouble sleeping, and depression among U.S. adults: A cross-sectional study from NHANES (2013–2016). BMC Public Health.

[28] Wu A.D., Gao M., Aveyard P., Taylor G. (2023). Smoking cessation and changes in anxiety and depression in adults with and without psychiatric disorders. JAMA Netw. Open.

[29] Kock L., Brown J., Cox S., McNeill A., Robson D., Shahab L., Tattan-Birch H., Brose L.S. (2023). Association of psychological distress with smoking cessation, duration of abstinence from smoking, and use of non-combustible nicotine-containing products: A cross-sectional population survey in Great Britain. Addict. Behav..

[30] De Boer N., Vermeulen J., Lin B., Van Os J., Ten Have M., De Graaf R., van Dorsselaer S., Bak M., Rutten B., Batalla A. (2023). Longitudinal associations between alcohol use, smoking, genetic risk scoring and symptoms of depression in the general population: A prospective 6-year cohort study. Psychol. Med..

[31] Incognito G.G., Grassi L., Palumbo M. (2024). Use of cigarettes and heated tobacco products during pregnancy and maternal–fetal outcomes: A retrospective, monocentric study. Arch. Gynecol. Obstet..

[32] Vysochyna I.L., Yashkina T.O., Kramarchuk V.V. (2025). Impact of electronic cigarette use on quality of life and autonomic nervous system function in young adults. Clin. Prev. Med..

[33] Lee B.G., Lee H., Kim N. (2025). Association between exclusive or dual use of combustible cigarettes and heated tobacco products and depressive symptoms. PLoS ONE.

[34] Heatherton T.F., Kozlowski L.T., Frecker R.C., Fagerström K.O. (1991). The Fagerström Test for Nicotine Dependence: A revision of the Fagerström Tolerance Questionnaire. Br. J. Addict..

[35] St. Helen G., Havel C., Dempsey D.A., Jacob P., Benowitz N.L. (2016). Nicotine delivery, retention and pharmacokinetics from various electronic cigarettes. Addiction.

[36] Lim W.M. (2023). Philosophy of science and research paradigm for business research in the transformative age of automation, digitalization, hyperconnectivity, obligations, globalization and sustainability. J. Trade Sci..

[37] Lim W.M., Khatri P., Shiva A., Dabas V. (2026). Guidelines for scale development and validation. J. Consum. Behav..

[38] Fekih-Romdhane F., Hallit R., Malaeb D., Sakr F., Dabbous M., Obeid S., Hallit S. (2025). Is it about substituting an addiction with another? Development and initial psychometric properties of the first heated tobacco products addiction questionnaire (HeaTPAQ). Addict. Sci. Clin. Pract..

[39] Spitzer R.L., Kroenke K., Williams J.B.W., Löwe B. (2006). A brief measure for assessing generalized anxiety disorder: The GAD-7. Arch. Intern. Med..

[40] Kroenke K., Spitzer R.L., Williams J.B.W. (2001). The PHQ-9: Validity of a brief depression severity measure. J. Gen. Intern. Med..

[41] Jamovi. https://www.jamovi.org/.

[42] Fluharty M., Taylor A.E., Grabski M., Munafò M.R. (2017). The association of cigarette smoking with depression and anxiety: A systematic review. Nicotine Tob. Res..

[43] Gouveia T.d.S., Pontes N.d.S., Lima M.B.P.d., Santos C.P., Ramos E.M.C., Christofaro D.G.D., Ramos D. (2022). Association between demographic factors, anxiety and depression in smokers. Cad. Saude Colet..

[44] Cho S., Park J.H., Kim D.H., Choi H., Park Y., Kim H.J., Lee A.-N., Shin J., Ha J. (2024). Association between combustible cigarettes and noncombustible nicotine or tobacco products and generalized anxiety disorder based on data from the 8th Korea National Health and Nutrition Examination Survey 2021. Korean J. Fam. Med..

[45] Seo Y.G., Lee S., Lim M.K., Paek Y.J. (2024). Heated tobacco product use and mental health: Korean National Health and Nutrition Examination Survey (2018–2020). Int. J. Ment. Health Addict..

[46] Nunes S.O.V., Vargas H.O., Prado E., Barbosa D.S., de Melo L.P., Moylan S., Dodd S., Berk M. (2013). The shared role of oxidative stress and inflammation in major depressive disorder and nicotine dependence. Neurosci. Biobehav. Rev..

[47] Addissouky T.A., El Sayed T., Ali M.M.A., Wang Y., El Baz A., Elarabany N., Khalil A.A. (2024). Oxidative stress and inflammation: Elucidating mechanisms of smoking-attributable pathology for therapeutic targeting. Bull. Natl. Res. Cent..

[48] Moylan S., Jacka F.N., Pasco J.A., Berk M. (2013). How cigarette smoking may increase the risk of anxiety symptoms and anxiety disorders: A critical review of biological pathways. Brain Behav..

[49] Bellosta S., Corsini A., Catena G. (2026). Heated tobacco product aerosol emission compared to cigarette smoke: A scoping review. Toxicol. Rep..

[50] Terlizzi E.P., Zablotsky B. (2024). Symptoms of Anxiety and Depression among Adults: United States, 2019 and 2022.

[51] Remes O., Francisco J., Templeton P. (2021). Biological, psychological, and social determinants of depression: A review of recent literature. Brain Sci..

[52] Lopresti A.L., Hood S.D., Drummond P.D. (2013). A review of lifestyle factors that contribute to important pathways associated with major depression: Diet, sleep and exercise. J. Affect. Disord..

